# GOSim – an R-package for computation of information theoretic GO similarities between terms and gene products

**DOI:** 10.1186/1471-2105-8-166

**Published:** 2007-05-22

**Authors:** Holger Fröhlich, Nora Speer, Annemarie Poustka, Tim Beißbarth

**Affiliations:** 1 German Cancer Research Center (DKFZ), Div. Molecular Genome Analysis, Im Neuenheimer Feld 580, 69120 Heidelberg, Germany; 2 Centre for Bioinformatics Tübingen (ZBIT), Sand 1, 72076 Tübingen, Germany

## Abstract

**Background:**

With the increased availability of high throughput data, such as DNA microarray data, researchers are capable of producing large amounts of biological data. During the analysis of such data often there is the need to further explore the similarity of genes not only with respect to their expression, but also with respect to their functional annotation which can be obtained from Gene Ontology (GO).

**Results:**

We present the freely available software package *GOSim*, which allows to calculate the functional similarity of genes based on various information theoretic similarity concepts for GO terms. *GOSim *extends existing tools by providing additional lately developed functional similarity measures for genes. These can e.g. be used to cluster genes according to their biological function. Vice versa, they can also be used to evaluate the homogeneity of a given grouping of genes with respect to their GO annotation. *GOSim *hence provides the researcher with a flexible and powerful tool to combine knowledge stored in GO with experimental data. It can be seen as complementary to other tools that, for instance, search for significantly overrepresented GO terms within a given group of genes.

**Conclusion:**

*GOSim *is implemented as a package for the statistical computing environment *R *and is distributed under GPL within the CRAN project.

## Background

Modern DNA microarray analysis has lead to an enormous collection of experimental data. Different analysis techniques are applied to gain insight into the underlying biological processes: statistical tests are used to find significantly regulated genes. Other methods cluster genes according to their expression profiles [1]. The hypothesis is that genes with expression patterns similar to known genes are involved in similar biological processes. In either case, researchers often end up with long lists of interesting candidate genes that need further examination. At this point, a second step is almost always applied: biologists categorize these genes to known biological functions and thus try to combine experimental outcomes with biological knowledge. Such information is e.g. provided by Gene Ontology (GO). GO has become one of the most widespread systems for systematically annotating gene products within the bioinformatics community and is developed by the Gene Ontology Consortium [2] with the intention to describe gene products with a controlled and structured vocabulary. GO terms are part of a Directed Acyclic Graph (DAG), covering three orthogonal taxonomies: *molecular function*, *biological process *and *cellular component*. Two different kinds of relationship between GO terms exist: the "is-a" relationship and the "part-of" relationship. By providing a standard vocabulary for all biological resources, GO enables researchers to use this information for further data analysis.

The *GOSim *package provides the user with an easy to use implementation of various information theoretical similarity concepts for GO terms [3-9]. It additionally implements different methods for computing functional similarities between gene products based on the similarities between the associated GO terms. This can for example be used for clustering genes according to their biological function [10,11] and thus may help to get a better understanding of the biological aspects covered by a set of genes. *GOSim *can be seen as complementary to existing tools that, for instance, search for significantly overrepresented GO terms within a given group of genes [12]. It extends methods like FuSSiMeg [8] by offering additional similarity measures for GO terms and gene products. With its specific focus, to our knowledge, *GOSim *is the most comprehensive software package of this kind.

## Methods

### GO term similarities

**Figure 1:**
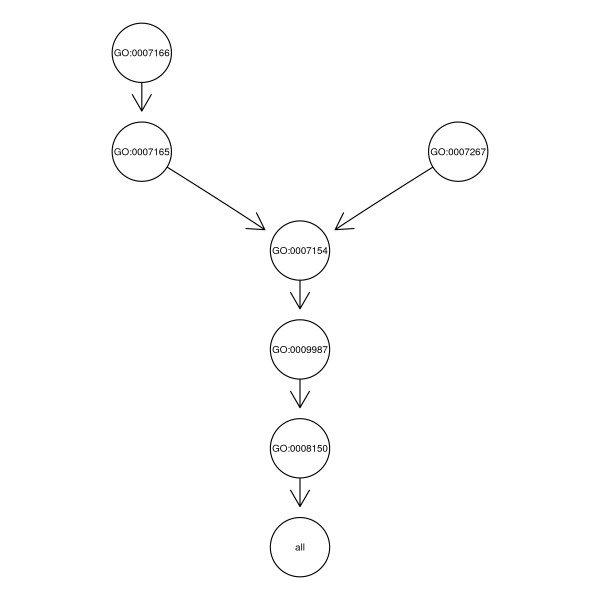
Example of a GO graph starting with leaves GO:0007166 and GO:0007267.

*GOSim *concentrates on similarity concepts for GO terms derived from information theory. One of the most well-known information theoretic similarity measures was introduced by Resnik [3]. It relies on the notion of the so-called *minimum subsumer *of two GO terms *t *and *t'*, which is the lowest common ancestor in the GO hierarchy (Figure 1). Its information content *IC*_*ms*_, which can be understood as a measure of similarity between *t *and *t'*, is given by:

sim(t,t′)=ICms(t,t′):=max⁡t^∈Pa(t,t′)IC(t^)
 MathType@MTEF@5@5@+=feaafiart1ev1aaatCvAUfKttLearuWrP9MDH5MBPbIqV92AaeXatLxBI9gBaebbnrfifHhDYfgasaacH8akY=wiFfYdH8Gipec8Eeeu0xXdbba9frFj0=OqFfea0dXdd9vqai=hGuQ8kuc9pgc9s8qqaq=dirpe0xb9q8qiLsFr0=vr0=vr0dc8meaabaqaciaacaGaaeqabaqabeGadaaakeaacqWGZbWCcqWGPbqAcqWGTbqBcqGGOaakcqWG0baDcqGGSaalcuWG0baDgaqbaiabcMcaPiabg2da9iabdMeajjabdoeadnaaBaaaleaacqWGTbqBcqWGZbWCaeqaaOGaeiikaGIaemiDaqNaeiilaWIafmiDaqNbauaacqGGPaqkcqGG6aGocqGH9aqpdaWfqaqaaiGbc2gaTjabcggaHjabcIha4bWcbaGafmiDaqNbaKaacqGHiiIZcqWGqbaucqWGHbqycqGGOaakcqWG0baDcqGGSaalcuWG0baDgaqbaiabcMcaPaqabaGccqWGjbqscqWGdbWqcqGGOaakcuWG0baDgaqcaiabcMcaPaaa@58D2@

Here *Pa*(*t*, *t'*) denotes the set of all common (also indirect) ancestors of GO terms *t *and *t'*, while *IC*(*t*) denotes the information content of term *t*. It is defined as (c.f. [7])

*IC*(*t*) = -log *P*(*t*)

i.e. as the negative logarithm of the probability of observing *t*. The information content of each GO term can be computed with *GOSim *for each of the taxonomies *molecular function*, *biological process *and *cellular component*. The calculation is based on counting, how many times a specific GO term or any of its direct or indirect offspring appear in annotated gene products. The association between gene products and GO identifiers is reported regularly by the GO Consortium [2]. The GO Consortium further provides evidence codes on the annotations, which can be used to calculate the information contents of all GO terms on a different basis. *GOSim *stores the information contents of all GO terms in data files to speed up all following computations. By default, for some combinations of evidence codes the information contents are already precomputed.

Besides Resnik's GO term similarity measure, extensions of Lin [5], and Jiang and Conrath [6] exist, which are included into *GOSim *as well. Both only differ in the way they normalize (Eq. 1). Jiang and Conrath's similarity measure is defined as

*sim*(*t*, *t*') = 1 - min(1, *IC*(*t*) - 2*IC*_*ms*_(*t*, *t*') + *IC*(*t*'))

i.e. the similarity between *t *and *t' *is 0, if their normalized distance is at least 1. Lin's pairwise similarity between GO terms is defined as:

sim(t,t′)=2ICms(t,t′)IC(t)+IC(t′)
 MathType@MTEF@5@5@+=feaafiart1ev1aaatCvAUfKttLearuWrP9MDH5MBPbIqV92AaeXatLxBI9gBaebbnrfifHhDYfgasaacH8akY=wiFfYdH8Gipec8Eeeu0xXdbba9frFj0=OqFfea0dXdd9vqai=hGuQ8kuc9pgc9s8qqaq=dirpe0xb9q8qiLsFr0=vr0=vr0dc8meaabaqaciaacaGaaeqabaqabeGadaaakeaacqWGZbWCcqWGPbqAcqWGTbqBcqGGOaakcqWG0baDcqGGSaalcuWG0baDgaqbaiabcMcaPiabg2da9maalaaabaGaeGOmaiJaemysaKKaem4qam0aaSbaaSqaaiabd2gaTjabdohaZbqabaGccqGGOaakcqWG0baDcqGGSaalcuWG0baDgaqbaiabcMcaPaqaaiabdMeajjabdoeadjabcIcaOiabdsha0jabcMcaPiabgUcaRiabdMeajjabdoeadjabcIcaOiqbdsha0zaafaGaeiykaKcaaaaa@4E9B@

*GOSim *also contains a similarity concept introduced by Couto et al. [9], which is not based on the minimum subsumer, but on the set of all so-called *disjunctive common ancestors*. Roughly speaking, the idea is not to consider the common ancestor having the highest information content only, but also others, if they are somehow "separate" from each other, i.e. there is a path to *t *and *t' *not passing any other of the disjunctive common ancestors. In our example from Figure 1 the set of disjunctive common ancestors only consists of the minimum subsumer, because any path from the other ancestors to GO:0007166 and GO:0007267 would have to pass the minimum subsumer. Based on the notion of disjunctive common ancestors Resnik's similarity concept can be extended by defining:

sim(t,t′)=ICshare(t,t′)=1|DisjCommAnc|∑t∈DisjCommAncIC(t)
 MathType@MTEF@5@5@+=feaafiart1ev1aaatCvAUfKttLearuWrP9MDH5MBPbIqV92AaeXatLxBI9gBaebbnrfifHhDYfgasaacH8akY=wiFfYdH8Gipec8Eeeu0xXdbba9frFj0=OqFfea0dXdd9vqai=hGuQ8kuc9pgc9s8qqaq=dirpe0xb9q8qiLsFr0=vr0=vr0dc8meaabaqaciaacaGaaeqabaqabeGadaaakeaacqWGZbWCcqWGPbqAcqWGTbqBcqGGOaakcqWG0baDcqGGSaalcuWG0baDgaqbaiabcMcaPiabg2da9iabdMeajjabdoeadnaaBaaaleaacqWGZbWCcqWGObaAcqWGHbqycqWGYbGCcqWGLbqzaeqaaOGaeiikaGIaemiDaqNaeiilaWIafmiDaqNbauaacqGGPaqkcqGH9aqpdaWcaaqaaiabigdaXaqaaiabcYha8jabdseaejabdMgaPjabdohaZjabdQgaQjabdoeadjabd+gaVjabd2gaTjabd2gaTjabdgeabjabd6gaUjabdogaJjabcYha8baadaaeqbqaaiabdMeajjabdoeadjabcIcaOiabdsha0jabcMcaPaWcbaGaemiDaqNaeyicI4SaemiraqKaemyAaKMaem4CamNaemOAaOMaem4qamKaem4Ba8MaemyBa0MaemyBa0MaemyqaeKaemOBa4Maem4yamgabeqdcqGHris5aaaa@71E3@

Likewise, Jiang-Conraths's and Lin's measures can be extended as well by replacing *IC*_*ms*_(*t*, *t'*) by *IC*_*share*_(*t*, *t'*). Finally, it should be mentioned that also the depth and density enriched term similarity by Couto et al. [8] has been integrated into *GOSim*.

### Functional gene similarities

The special strength of *GOSim *lies in the possibility not only to calculate similarities for individual GO terms, but also for genes based on their complete GO annotation. For this purpose three basic ideas have been implemented:

1. Computation of the maximum and average similarity between any pair of GO terms.

2. Computation of a so-called *optimal assignment *of terms from one gene to those of another one [11].

3. Embedding of each gene into a feature space defined by the gene's similarity to certain prototype genes [10,11]. Within this feature space similarities naturally arise as dot products between the feature vectors. These dot products can be understood as so-called *kernel functions*, as used e.g. in Support Vector Machines [13].

Especially the inclusion of the last two methods clearly distinguishes *GOSim *from existing tools like FuSSiMeg [8]. More information on these methods, including a detailed evaluation, can also be found in our earlier publications [10] and [11].

#### Maximum and average pairwise GO term similarity

The idea of the maximum pairwise GO term similarity is straight forward and is for instance employed in FuSSiMeg [8]. Given two genes *g *and *g' *annotated with GO terms *t*_1_,..., *t*_*n *_and t′1,...,t′m
 MathType@MTEF@5@5@+=feaafiart1ev1aaatCvAUfKttLearuWrP9MDH5MBPbIqV92AaeXatLxBI9gBaebbnrfifHhDYfgasaacH8akY=wiFfYdH8Gipec8Eeeu0xXdbba9frFj0=OqFfea0dXdd9vqai=hGuQ8kuc9pgc9s8qqaq=dirpe0xb9q8qiLsFr0=vr0=vr0dc8meaabaqaciaacaGaaeqabaqabeGadaaakeaacuWG0baDgaqbamaaBaaaleaacqaIXaqmaeqaaOGaeiilaWIaeiOla4IaeiOla4IaeiOla4IaeiilaWIafmiDaqNbauaadaWgaaWcbaGaemyBa0gabeaaaaa@36C7@ we define the functional similarity between between *g *and *g' *as

simgene(g,g′)=max⁡i=1,,...,nj=1,...,msim(ti,t′j)
 MathType@MTEF@5@5@+=feaafiart1ev1aaatCvAUfKttLearuWrP9MDH5MBPbIqV92AaeXatLxBI9gBaebbnrfifHhDYfgasaacH8akY=wiFfYdH8Gipec8Eeeu0xXdbba9frFj0=OqFfea0dXdd9vqai=hGuQ8kuc9pgc9s8qqaq=dirpe0xb9q8qiLsFr0=vr0=vr0dc8meaabaqaciaacaGaaeqabaqabeGadaaakeaacqWGZbWCcqWGPbqAcqWGTbqBdaWgaaWcbaGaem4zaCMaemyzauMaemOBa4MaemyzaugabeaakiabcIcaOiabdEgaNjabcYcaSiqbdEgaNzaafaGaeiykaKIaeyypa0ZaaCbeaeaadaWfqaqaaiGbc2gaTjabcggaHjabcIha4bWcbaGaemyAaKMaeyypa0JaeGymaeJaeiilaWIaeiilaWIaeiOla4IaeiOla4IaeiOla4IaeiilaWIaemOBa4gabeaaaeaacqWGQbGAcqGH9aqpcqaIXaqmcqGGSaalcqGGUaGlcqGGUaGlcqGGUaGlcqGGSaalcqWGTbqBaeqaaOGaem4CamNaemyAaKMaemyBa0MaeiikaGIaemiDaq3aaSbaaSqaaiabdMgaPbqabaGccqGGSaalcuWG0baDgaqbamaaBaaaleaacqWGQbGAaeqaaOGaeiykaKcaaa@614F@

where *sim *is some similarity measure to compare GO terms *t*_*i *_and t′j
 MathType@MTEF@5@5@+=feaafiart1ev1aaatCvAUfKttLearuWrP9MDH5MBPbIqV92AaeXatLxBI9gBaebbnrfifHhDYfgasaacH8akY=wiFfYdH8Gipec8Eeeu0xXdbba9frFj0=OqFfea0dXdd9vqai=hGuQ8kuc9pgc9s8qqaq=dirpe0xb9q8qiLsFr0=vr0=vr0dc8meaabaqaciaacaGaaeqabaqabeGadaaakeaacuWG0baDgaqbamaaBaaaleaacqWGQbGAaeqaaaaa@2FB2@. In *GOSim *the resulting value can be further normalized to account for an unequal number of GO terms for both genes:

simgene(g,g′)←simgene(g,g′)simgene(g,g)simgene(g′,g′)
 MathType@MTEF@5@5@+=feaafiart1ev1aaatCvAUfKttLearuWrP9MDH5MBPbIqV92AaeXatLxBI9gBaebbnrfifHhDYfgasaacH8akY=wiFfYdH8Gipec8Eeeu0xXdbba9frFj0=OqFfea0dXdd9vqai=hGuQ8kuc9pgc9s8qqaq=dirpe0xb9q8qiLsFr0=vr0=vr0dc8meaabaqaciaacaGaaeqabaqabeGadaaakeaacqWGZbWCcqWGPbqAcqWGTbqBdaWgaaWcbaGaem4zaCMaemyzauMaemOBa4MaemyzaugabeaakiabcIcaOiabdEgaNjabcYcaSiqbdEgaNzaafaGaeiykaKIaeyiKHW6aaSaaaeaacqWGZbWCcqWGPbqAcqWGTbqBdaWgaaWcbaGaem4zaCMaemyzauMaemOBa4MaemyzaugabeaakiabcIcaOiabdEgaNjabcYcaSiqbdEgaNzaafaGaeiykaKcabaWaaOaaaeaacqWGZbWCcqWGPbqAcqWGTbqBdaWgaaWcbaGaem4zaCMaemyzauMaemOBa4MaemyzaugabeaakiabcIcaOiabdEgaNjabcYcaSiabdEgaNjabcMcaPiabdohaZjabdMgaPjabd2gaTnaaBaaaleaacqWGNbWzcqWGLbqzcqWGUbGBcqWGLbqzaeqaaOGaeiikaGIafm4zaCMbauaacqGGSaalcuWGNbWzgaqbaiabcMcaPaWcbeaaaaaaaa@6B04@

Instead of computing the maximum pairwise GO term similarity one may also take the average here.

#### Optimal assignment gene similarities

**Figure 2:**
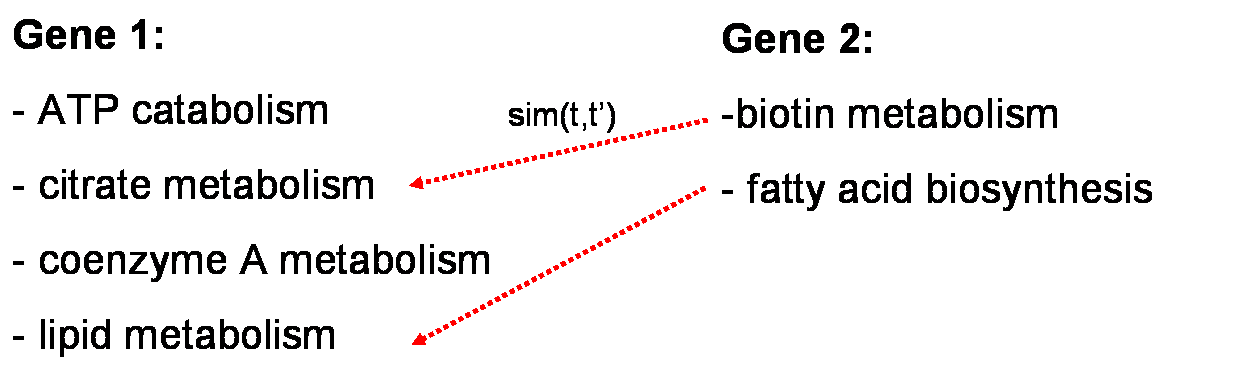
Idea of an optimal assignment: each GO term belonging to gene 2 is assigned to exactly one GO term belonging to gene 1 such that the overall GO term similarity is maximized.

Given a similarity concept *sim *to compare individual GO terms, the idea of an optimal assignment is to assign each term of the gene having fewer GO terms to exactly one term of the other gene such that the overall similarity is maximized (c.f. Figure 2). More formally this can be stated as follows: Let *π *be some permutation of either an *n*-subset of natural numbers {1,..., *m*} or an *m*-subset of natural numbers {1,..., *n*} (this will be clear from context). Then we are looking for the quantity

simgene(g,g′)={max⁡π∑i=1nsim(ti,t′π(i))if m>nmax⁡π∑j=1msim(tπ(j),t′j)otherwise
 MathType@MTEF@5@5@+=feaafiart1ev1aaatCvAUfKttLearuWrP9MDH5MBPbIqV92AaeXatLxBI9gBaebbnrfifHhDYfgasaacH8akY=wiFfYdH8Gipec8Eeeu0xXdbba9frFj0=OqFfea0dXdd9vqai=hGuQ8kuc9pgc9s8qqaq=dirpe0xb9q8qiLsFr0=vr0=vr0dc8meaabaqaciaacaGaaeqabaqabeGadaaakeaacqWGZbWCcqWGPbqAcqWGTbqBdaWgaaWcbaGaem4zaCMaemyzauMaemOBa4MaemyzaugabeaakiabcIcaOiabdEgaNjabcYcaSiqbdEgaNzaafaGaeiykaKIaeyypa0ZaaiqaaeaafaqaaeGacaaabaGagiyBa0MaeiyyaeMaeiiEaG3aaSbaaSqaaGGaciab=b8aWbqabaGcdaaeWaqaaiabdohaZjabdMgaPjabd2gaTjabcIcaOiabdsha0naaBaaaleaacqWGPbqAaeqaaOGaeiilaWIafmiDaqNbauaadaWgaaWcbaGae8hWdaNaeiikaGIaemyAaKMaeiykaKcabeaakiabcMcaPaWcbaGaemyAaKMaeyypa0JaeGymaedabaGaemOBa4ganiabggHiLdaakeaaieaacqGFPbqAcqGFMbGzcqqGGaaicqWGTbqBcqGH+aGpcqWGUbGBaeaacyGGTbqBcqGGHbqycqGG4baEdaWgaaWcbaGae8hWdahabeaakmaaqadabaGaem4CamNaemyAaKMaemyBa0MaeiikaGIaemiDaq3aaSbaaSqaaiab=b8aWjabcIcaOiabdQgaQjabcMcaPaqabaGccqGGSaalcuWG0baDgaqbamaaBaaaleaacqWGQbGAaeqaaOGaeiykaKcaleaacqWGQbGAcqGH9aqpcqaIXaqmaeaacqWGTbqBa0GaeyyeIuoaaOqaaiab+9gaVjab+rha0jab+HgaOjab+vgaLjab+jhaYjab+Dha3jab+LgaPjab+nhaZjab+vgaLbaaaiaawUhaaaaa@8BB8@

The computation of (Eq. 8) corresponds to the solution of the classical maximum weighted bipartite matching (optimal assignment) problem in graph theory and can be carried out in *O*(max(*n*, *m*)^3^) time [14]. To prevent that larger lists of terms automatically achieve a higher similarity we should again normalize *sim*_*gene *_according to (Eq. 7).

#### Feature space embedding of gene products

The idea of this method is to calculate for each gene *g *feature vectors *φ*(*g*) by using their similarity to certain prototype genes *p*_1_,..., *p*_*n*_:

*φ*(*g*) = (*sim'*(*g*, *p*_1_),..., *sim*'(*g*, *p*_*n*_))^*T*^

By default the 250 best annotated genes, i.e. which have been annotated with GO terms most often, are used as prototypes, and *sim' *is the maximum pairwise GO term similarity. Alternatively, one can use the optimal assignment similarity for *sim' *as well. Both similarity measures can by itself again be combined with arbitrary GO term similarity concepts. The default is that of Jiang and Conrath.

Feature space constructions like in (Eq. 9) are known from the literature on Support Vector Machines and other kernel methods and give rise to so-called "empirical kernel maps" [13].

**Figure 3:**
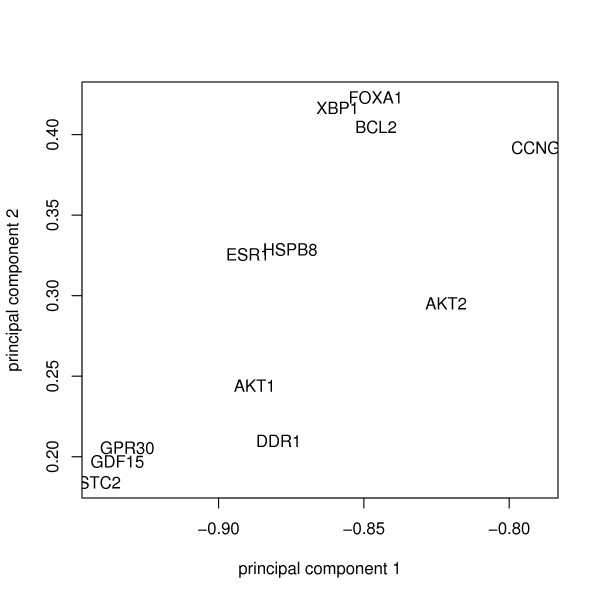
Genes embedded into a feature space defined by the GO similarity to certain prototype genes. principal components analysis was used to reduce the dimensionality of the feature space and the first two principal components are displayed.

Because the feature vectors are very high-dimensional we usually perform a principal component analysis (PCA) to project the data into a lower dimensional subspace (Figure 3). The number of principal components is by default chosen such that at least 95% of the total variance in feature space can be explained (this is a relatively conservatve criterion), and the feature vectors are normalized to norm 1. It should be mentioned that in principle one can combine functional similarities between gene products with regard to different GO sub-categories ("biological process", "molecular function", "cellular component"). An obvious way for doing so would be to consider the sum of the respective similarities:

*sim*_*total*_(*g*, *g*') = *sim*_*Ontology*1_(*g*, *g*') + *sim*_*Ontology*2_(*g*, *g*')

Of course, one could also use a weighted averaging scheme here, if desired.

#### Functional gene clustering

The calculated GO similarities between gene products can be used to cluster genes with respect to their function. The practical usage of this method is highlighted in more detail in an example study on microarray data in the Results Section of this paper.

#### Cluster evaluations

*GOSim *has the possibility to evaluate a given clustering of genes or terms by means of their GO similarities. Supposed we have decided to group genes into certain clusters on the basis of other experiments (e.g. microarray). Then we can ask ourselves, how similar these groups are with respect to their GO annotations. *GOSim *uses the functional similarity between genes to calculate for each cluster the median within cluster similarity and the median absolute deviation (mad). Moreover, a visualization via cluster silhouettes [15] is provided by *GOSim *as well. Likewise, different groupings of GO terms can be evaluated in a similar manner. Again, the practical usage of this method is highlighted in more detail in the example study contained in the Results Section.

## Implementation

*GOSim *is implemented as a package within the statistical computing environment *R *and is distributed under GPL within the CRAN project [16]. Functions of the following *R *packages are internally utilized and are hence required by *GOSim *to work properly:

• *GOStats *(≥ 1.7)

• *mclust *(≥ 2.1)

• *cluster *(≥ 1.11)

Furthermore, *Rgraphviz *is recommended to visualize GO graphs. To calculate functional similarities between gene products *GOSim *needs their Entrez Gene IDs. The mapping of these IDs to the Gene Ontology is provided by the *R *package *GO*, which is required by the *GOStats *package. Genes without annotation are filtered out automatically, when *GOSim *performs similarity calculations for gene products. Their exists a function in *GOSim *to specify, on which sub-category ("biological process", "molecular function", "cellular component") all computations are based on. Moreover, it is possible to restrict the GO annotation of gene products to arbitrary user defined evidence codes.

To summarize, *GOSim *provides *R*-methods for the following tasks:

• low-level functions for GO graph traversal

• specification of the GO sub-category ("biological process", "molecular function", "cellular component") and of evidence codes

• calculation of the information content of GO terms and of the similarity between GO terms (see last Section)

• similarity calculation between gene products based on their GO annotations (see last Section)

• filtering and printing the GO annotation of a given list of genes

• evaluation of a given clustering of genes or terms via precomputed similarities (see last Section)

## Results

### Data

In this Section we show an example application of the *GOSim *package to an analysis of DNA microarray data. The data was taken from [17], who derived a gene expression profile for dilated cardiomyopathy based on cDNA and Affymetrix microarray chips. The details of this study can be found in the paper. The number of differentially upregulated genes was 1107 on the cDNA and 336 on the Affymetrix microarray data. The number of differentially downregulated genes was 278 on the cDNA and 67 on the Affymetrix chips.

### Methods

The Entrez Gene IDs of differentially upregulated and downregulated genes collected from the study were treated separately for cDNA and Affymetrix chips. 677 of the differentially upregulated genes on the cDNA chips and 230 on the Affymetrix chips showed a mapping to the GO category "biological process". 157 differentially downregulated genes could be mapped on the cDNA and 43 on the Affymetrix chips. We used the *GOSim *package to calculate gene similarities based on the feature vector representation [10,11] (c.f. Section Background). This was done by defining each gene by its maximum Jiang-Conrath similarity [6] to 250 prototypes genes. Prototype genes were those 250 genes, which were most frequently annotated with GO terms. The similarity between feature vectors *x*, *y *was taken as their normalized dot product (c.f. Section Background):

sim(x,y)=〈x,y〉‖x‖‖y‖
 MathType@MTEF@5@5@+=feaafiart1ev1aaatCvAUfKttLearuWrP9MDH5MBPbIqV92AaeXatLxBI9gBaebbnrfifHhDYfgasaacH8akY=wiFfYdH8Gipec8Eeeu0xXdbba9frFj0=OqFfea0dXdd9vqai=hGuQ8kuc9pgc9s8qqaq=dirpe0xb9q8qiLsFr0=vr0=vr0dc8meaabaqaciaacaGaaeqabaqabeGadaaakeaacqWGZbWCcqWGPbqAcqWGTbqBcqGGOaakcqWG4baEcqGGSaalcqWG5bqEcqGGPaqkcqGH9aqpdaWcaaqaaiabgMYiHlabdIha4jabcYcaSiabdMha5jabgQYiXdqaamaafmaabaGaemiEaGhacaGLjWUaayPcSdWaauWaaeaacqWG5bqEaiaawMa7caGLkWoaaaaaaa@480E@

Prior to similarity calculation we performed a principal component analysis (PCA) on the feature vectors to reduce their dimensionality. The number of principal components was chosen such that at least 95% of the total variance in feature space could be explained (c.f. Section "Feature Space Embedding of Gene Products"). This way we obtained 29 principal components for the upregulated and 23 principal components for the downregulated genes on the cDNA chips. For the Affymetrix chips the number of principal components was 24 for the upregulated and 11 for the downregulated genes.

**Figure 4:**
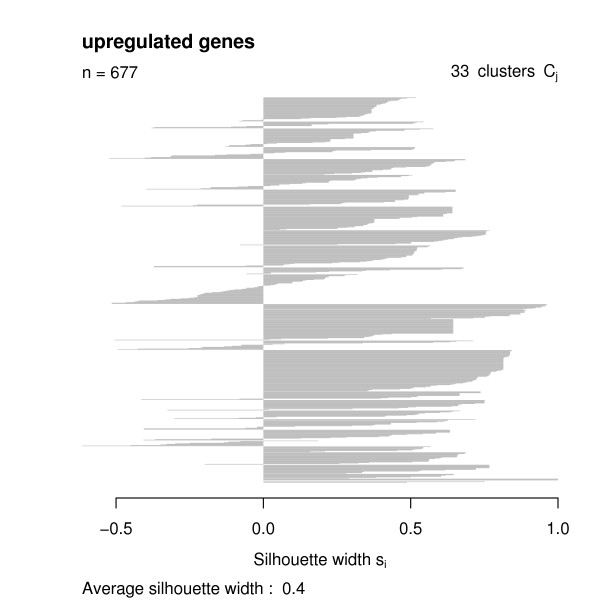
Clustering silhouette of the upregulated genes (cDNA chips).

**Figure 5:**
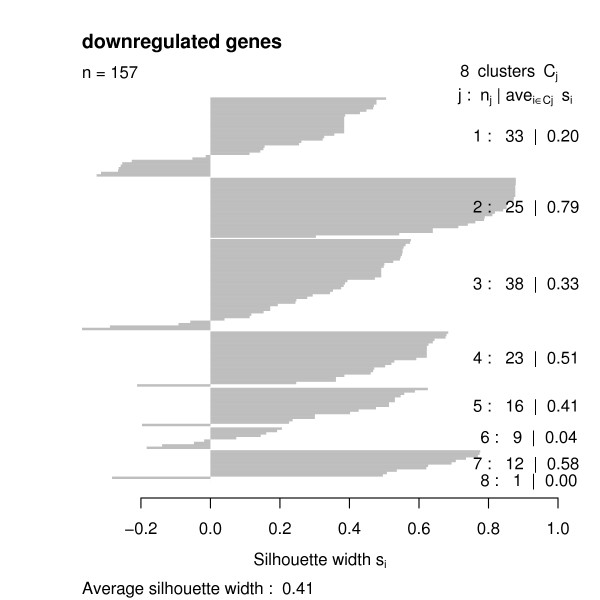
Clustering silhouette of the downregulated genes (cDNA chips).

**Figure 6:**
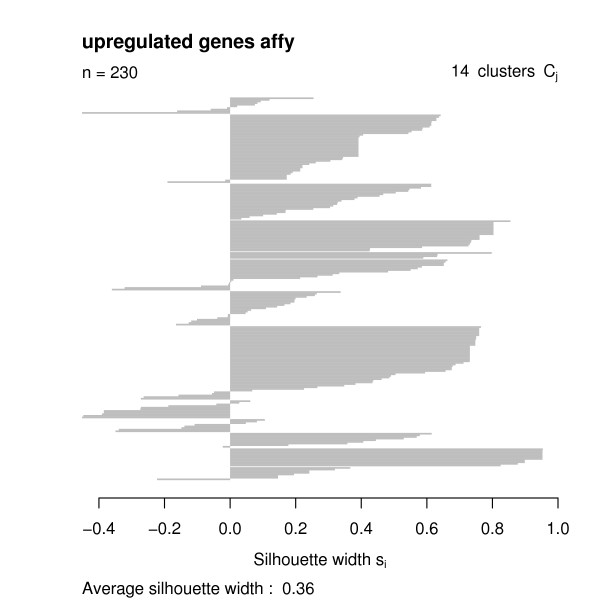
Clustering silhouette of the upregulated genes (Affymetrix chips).

**Figure 7:**
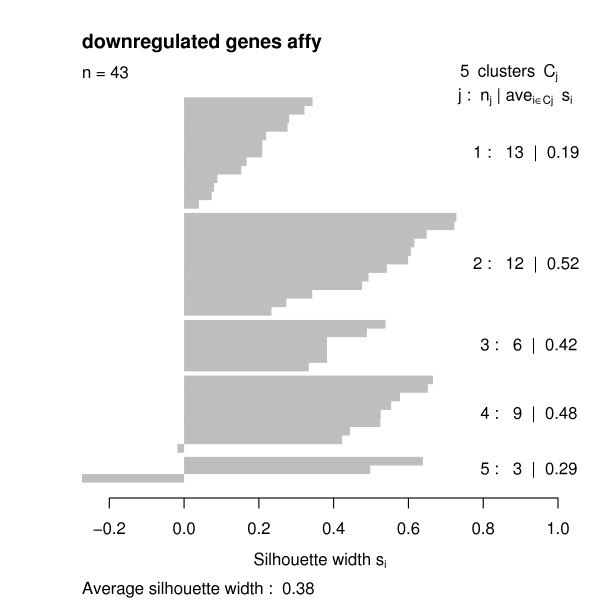
Clustering silhouette of the downregulated genes (Affymetrix chips).

Having the functional similarities between regulated genes, we used the *GOSim *package to compute a hierarchical clustering using Ward's method. We decided to cut the clustering tree at height 0.05 and looked at the clustering silhouettes by employing the *GOSim *package (Figures 4, 5, 6, 7). Cluster silhouettes [15] are a classical way of depicting the quality of a given clustering of objects. The silhouette value for each point in a cluster is a measure of how similar that point is to points in its own cluster vs. points in other clusters, and ranges from -1 to +1. It is defined as:

S(i)=min⁡j(d¯B(i,j))−d¯W(i)max⁡(d¯W(i),min⁡j(d¯B(i,j))
 MathType@MTEF@5@5@+=feaafiart1ev1aaatCvAUfKttLearuWrP9MDH5MBPbIqV92AaeXatLxBI9gBaebbnrfifHhDYfgasaacH8akY=wiFfYdH8Gipec8Eeeu0xXdbba9frFj0=OqFfea0dXdd9vqai=hGuQ8kuc9pgc9s8qqaq=dirpe0xb9q8qiLsFr0=vr0=vr0dc8meaabaqaciaacaGaaeqabaqabeGadaaakeaacqWGtbWucqGGOaakcqWGPbqAcqGGPaqkcqGH9aqpdaWcaaqaaiGbc2gaTjabcMgaPjabc6gaUnaaBaaaleaacqWGQbGAaeqaaOGaeiikaGIafmizaqMbaebadaWgaaWcbaGaemOqaieabeaakiabcIcaOiabdMgaPjabcYcaSiabdQgaQjabcMcaPiabcMcaPiabgkHiTiqbdsgaKzaaraWaaSbaaSqaaiabdEfaxbqabaGccqGGOaakcqWGPbqAcqGGPaqkaeaacyGGTbqBcqGGHbqycqGG4baEcqGGOaakcuWGKbazgaqeamaaBaaaleaacqWGxbWvaeqaaOGaeiikaGIaemyAaKMaeiykaKIaeiilaWIagiyBa0MaeiyAaKMaeiOBa42aaSbaaSqaaiabdQgaQbqabaGccqGGOaakcuWGKbazgaqeamaaBaaaleaacqWGcbGqaeqaaOGaeiikaGIaemyAaKMaeiilaWIaemOAaOMaeiykaKIaeiykaKcaaaaa@6349@

where d¯
 MathType@MTEF@5@5@+=feaafiart1ev1aaatCvAUfKttLearuWrP9MDH5MBPbIqV92AaeXatLxBI9gBaebbnrfifHhDYfgasaacH8akY=wiFfYdH8Gipec8Eeeu0xXdbba9frFj0=OqFfea0dXdd9vqai=hGuQ8kuc9pgc9s8qqaq=dirpe0xb9q8qiLsFr0=vr0=vr0dc8meaabaqaciaacaGaaeqabaqabeGadaaakeaacuWGKbazgaqeaaaa@2E15@_*W*_(*i*) is the average distance from the i-th point to the other points in its own cluster, and d¯
 MathType@MTEF@5@5@+=feaafiart1ev1aaatCvAUfKttLearuWrP9MDH5MBPbIqV92AaeXatLxBI9gBaebbnrfifHhDYfgasaacH8akY=wiFfYdH8Gipec8Eeeu0xXdbba9frFj0=OqFfea0dXdd9vqai=hGuQ8kuc9pgc9s8qqaq=dirpe0xb9q8qiLsFr0=vr0=vr0dc8meaabaqaciaacaGaaeqabaqabeGadaaakeaacuWGKbazgaqeaaaa@2E15@_*B*_(*i*, *j*) is the average distance from the i-th point to points in another cluster j. The quality for a given cluster can effectively be expressed by the average silhouette value (= cluster silhouette index) of points belonging to that specific cluster.

### Analysis results

The clustering silhouettes for up- and downregulated genes show that there exists several functional groups with silhouette cluster index greater 0.5 (upregulated genes in clusters 9, 13, 14, 15, 16, 18, 20, 23, 28, 29, 30, 31, 33 on the cDNA platform, upregulated genes in clusters 4, 5, 8, 12 on the Affymetrix platform, downregulated genes in clusters 2, 4, 7 on the cDNA chips, downregulated genes in cluster 2 on the Affymetrix platform – c.f. Table 1). Upregulated genes on the cDNA chips are involved into various forms of transport (clusters 9, 13), into the intracellular signaling cascade (cluster 13), cell adhesion (cluster 14), transcription (clusters 15, 18), signal transduction (cluster 15), cytoskeleton organization and biogenesis (cluster 16), immune response (cluster 20), catabolism (cluster 23), protein localization (cluster 28), protein modification (cluster 29), chromatin assembly or disassembly (cluster 30), mitochondrial electron transport (cluster 31) and fibroblast growth factor receptor signaling pathway (cluster 33). Upregulated genes on the Affymetrix chips are involved into metabolism and transport (cluster 4) blood coagulation and immune response (cluster 5), transcription (cluster 8) and cell adhesion (cluster 12).

**Table 1:** Functional groups found on the different chip platforms.

**Clust. no.**	**Clust. size**	**sil. ind.**	**platform**	**regulation**	**function**
9	27	0.62	cDNA	up	transport
13	10	0.93	cDNA	up	intracellular signaling cascade, protein transport
14	16	0.82	cDNA	up	cell adhesion
15	38	0.51	cDNA	up	transcription, signal transduction
16	5	0.58	cDNA	up	cytoskeleton organization and biogenesis, behavior
18	74	0.74	cDNA	up	transcription
20	15	0.67	cDNA	up	immune response
23	2	0.67	cDNA	up	catabolism
28	22	0.54	cDNA	up	protein localization
29	16	0.61	cDNA	up	protein modification
30	8	0.54	cDNA	up	chromatin assembly or disassembly, protein complex assembly
31	2	1.0	cDNA	up	mitochondrial electron transport, NADH to ubiquinone
33	2	0.62	cDNA	up	fibroblast growth factor receptor signaling pathway
2	25	0.79	cDNA	down	transcription
4	23	0.51	cDNA	down	transport
7	12	0.58	cDNA	down	immune response
4	19	0.73	Affymetrix	up	metabolism, transport
5	4	0.66	Affymetrix	up	blood coagulation, immune response
8	45	0.54	Affymetrix	up	transcription
12	11	0.92	Affymetrix	up	cell adhesion
2	12	0.52	Affymetrix	down	metabolism

In contrast, downregulated genes on the cDNA chips are involved into transcription (cluster 2), transport (cluster 4) and immune response (cluster 7). On the Affymetrix chips there is just one identifiable cluster of downregulated genes are involved into metabolism.

The whole clusters are also included in an additional supplement as text files. In conclusion of this analysis one could say that in this data upregulated genes are more involved in protein modification, protein localization, blood coagulation and cell adhesion while downregulated genes are more related to metabolic tasks.

These findings can be seen complementary to those by Bart et al., who found a significant enrichment of GO terms related to protein biosynthesis in upregulated and GO terms related to immune response in downregulated genes. Both results are based on different statistical analysis methods of the data. Functional gene clustering is based on comparing the full GO term profiles for genes of interest, while GO-significance analysis looks for the statistical over-representation of individual GO terms in the full list of regulated genes. An advantage of a functional gene clustering compared to a traditional GO-significance analysis is that the complete list of regulated genes is structured into functionally related groups, which can help later interpretation. This kind of analysis is possible for very large as well as for very small lists. On the other hand functional clusters may also contain several genes, which are regarded as statistical significant, but are actually not differentially expressed (false positives). Hence, the interpretation of small functional clusters should generally be taken with care.

## Discussion

The *GOSim *package offers an easy and straight forward way to gain insights into functional gene groups by using the Gene Ontology. It offers a rich toolbox of similarity concepts for GO terms as well as for gene products. The present example study highlights the usefulness of *GOSim *in a real life scenario.

## Conclusion

The *GOSim *package integrates information theoretic similarity concepts for GO terms and derived functional similarity measures for gene products in a novel and unique way. Applications thereof are clusterings of gene products with regard to their function [10,11] or scorings of a given grouping of genes or terms with regard to their GO similarities. Both tasks can be performed with *GOSim *in a simple and straight forward way. It hence provides the user with a flexible and powerful tool to combine biological knowledge with experimental data. *GOSim *systematically extends existing tools like FuSSiMeg [8] by integrating its functionality and providing additional similarity concepts for gene products [10,11]. *GOSim *is implemented as a package for the statistical computing environment *R *and has been integrated into the CRAN project. It is thus made available for a broad community of potential users. More documentation on the implemented methods can be found in the user manual. Detailed quantitative results can be found in [8-11].

## Availability and requirements

• **Project name**: *GOSim*

• **Project home page**: 

• **Operating system(s)**: platform independent

• **Programming language**: *R*

• **License**: GPL

The software is also included as a supplement to this paper.

## Authors' contributions

The feature space embedding and the optimal assignment similarity were invented by HF and NS. The *R*-implementation of *GOSim *was carried out by HF. Substantial advice and suggestions were given by TB and AP. All authors read and approved the final manuscript.

## Supplementary Material

Additional File 1 Clustering results. Clustering results for the example study in the Results Section. Click here for file

Additional File 2 *GOSim *version 1.0. The *GOSim *software package. Click here for file
