# Supplementary figures and images for: GOSim – an R-package for computation of information theoretic GO similarities between terms and gene products
Source: BMC Bioinformatics. 2007 May 22;8:166. doi: 10.1186/1471-2105-8-166 (PMC1892785; doi:10.1186/1471-2105-8-166)

# Cluster Dendrogram

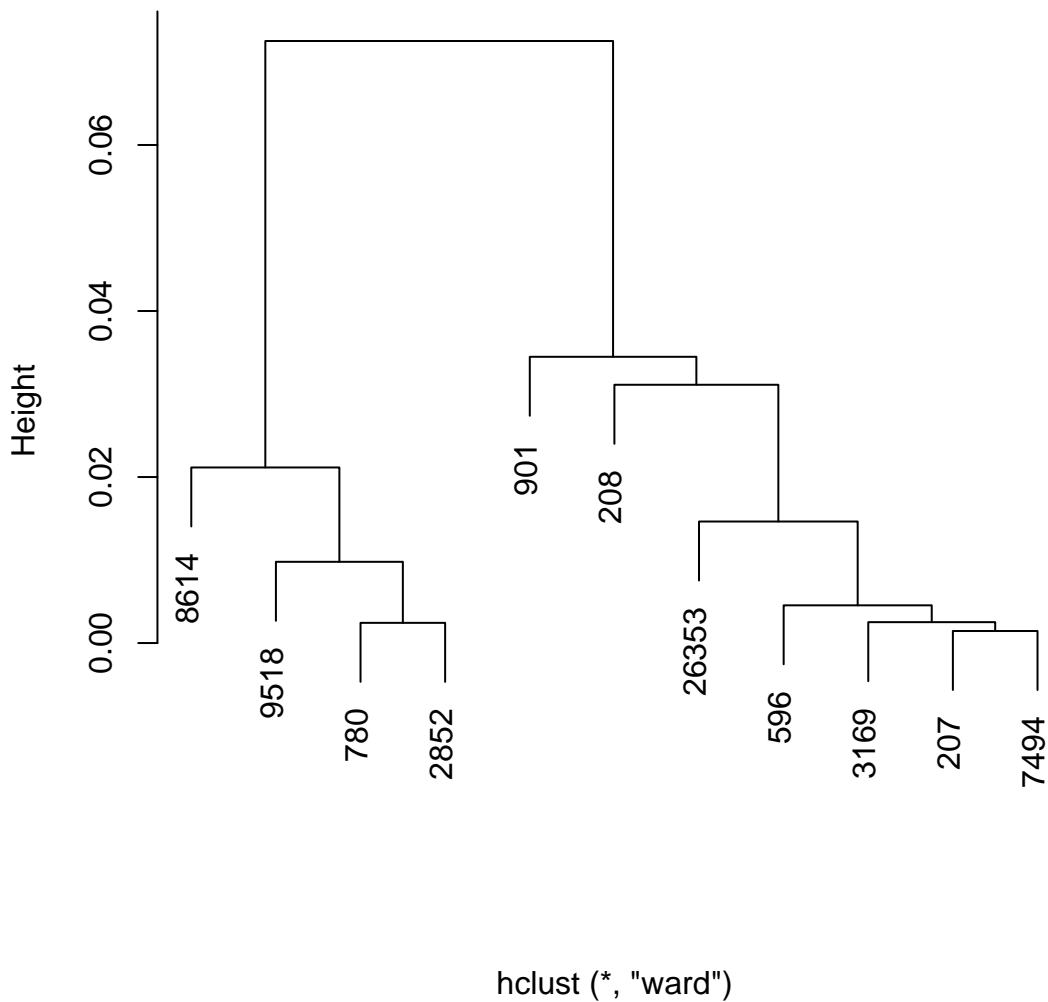

Supplement: Additional File 2 — GOSim version 1.0. The GOSim software package. [file 1471-2105-8-166-S2.gz › GOSim/inst/doc/GOClusterExample.pdf]

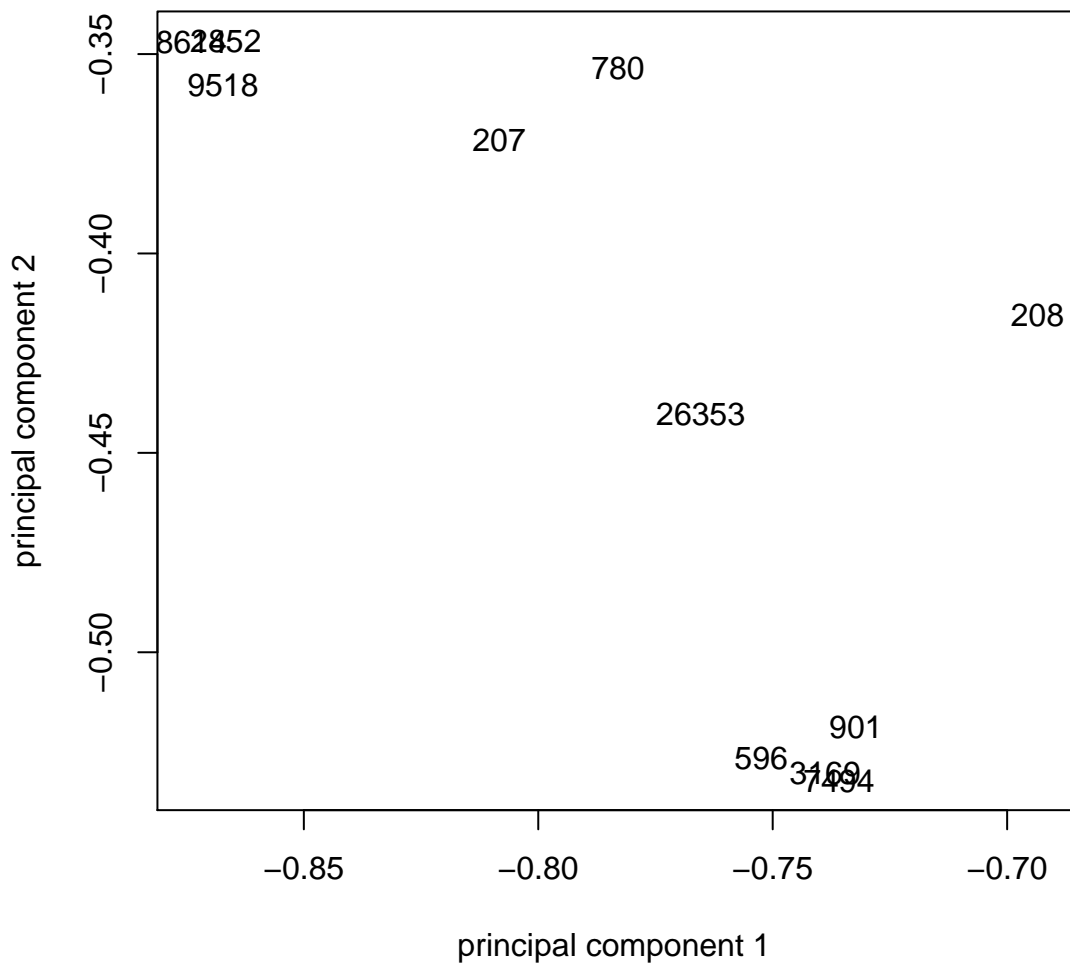

Supplement: Additional File 2 — GOSim version 1.0. The GOSim software package. [file 1471-2105-8-166-S2.gz › GOSim/inst/doc/GOPCAExample.pdf]

n = 11

3 clusters  $C_j$

$j : n_j \mid \text{ave}_{i \in C_j} s_i$

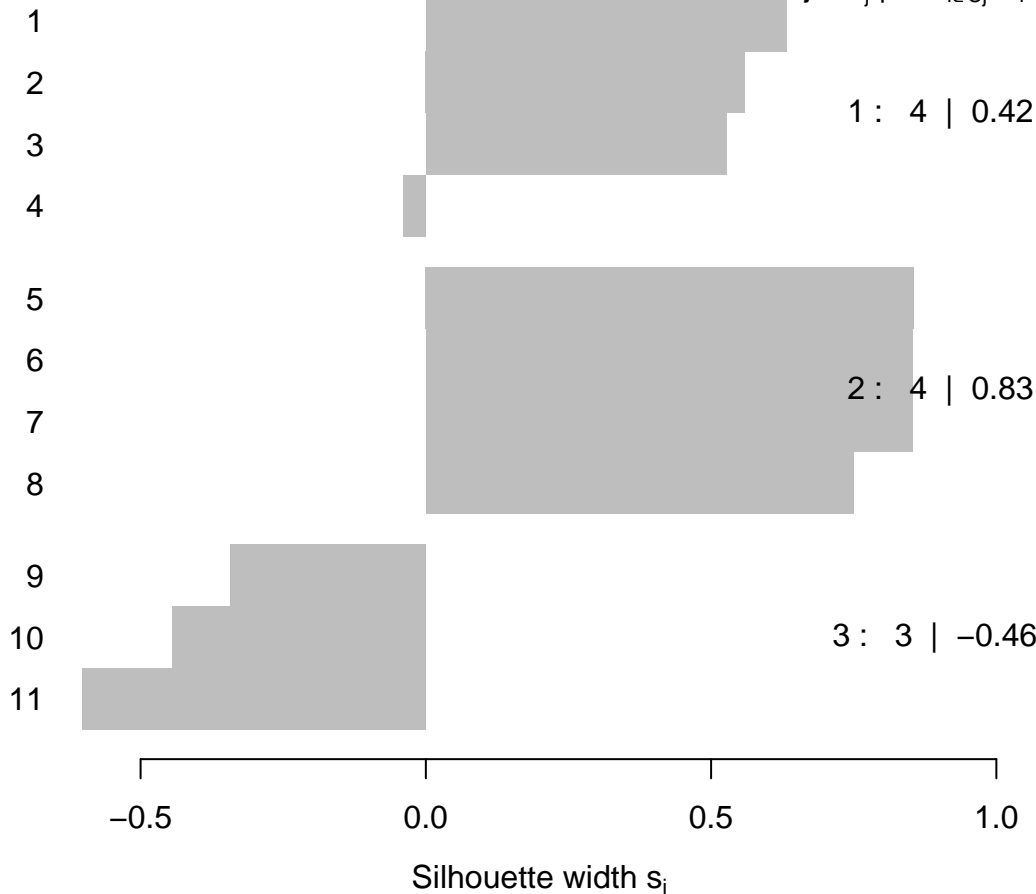

Supplement: Additional File 2 — GOSim version 1.0. The GOSim software package. [file 1471-2105-8-166-S2.gz › GOSim/inst/doc/GOClustersil.pdf]

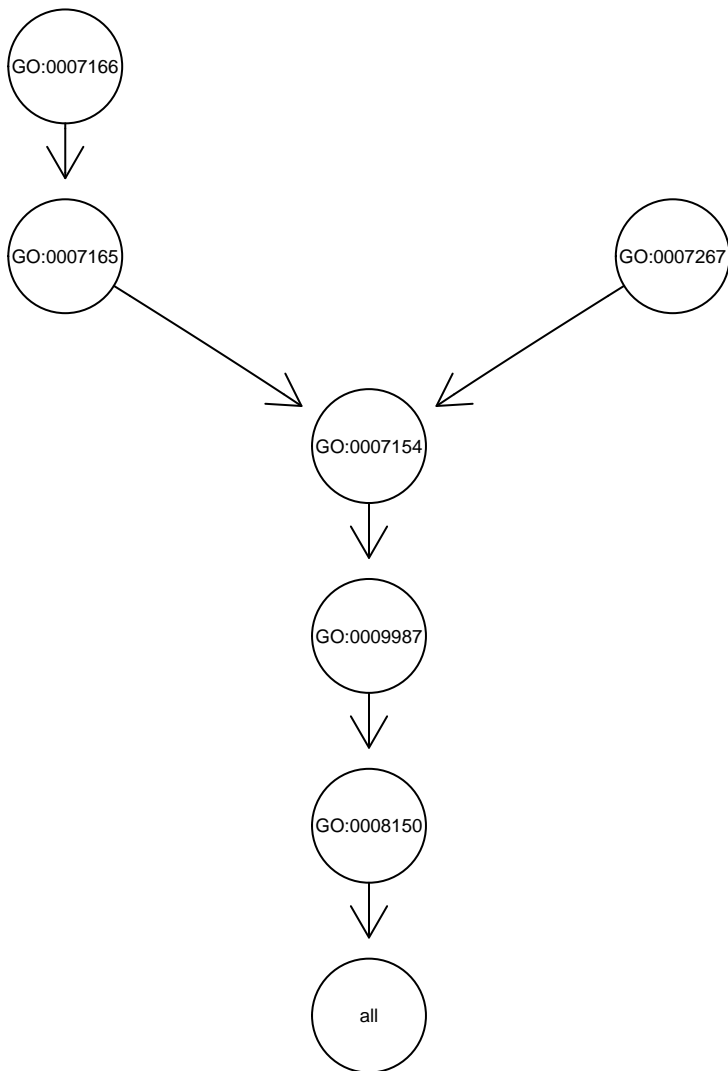

Supplement: Additional File 2 — GOSim version 1.0. The GOSim software package. [file 1471-2105-8-166-S2.gz › GOSim/inst/doc/gosim-GOExample.pdf]
